# Colchicine Blocks Tubulin Heterodimer Recycling by Tubulin Cofactors TBCA, TBCB, and TBCE

**DOI:** 10.3389/fcell.2021.656273

**Published:** 2021-04-22

**Authors:** Sofia Nolasco, Javier Bellido, Marina Serna, Bruno Carmona, Helena Soares, Juan Carlos Zabala

**Affiliations:** ^1^Faculdade de Medicina Veterinária, CIISA – Centro de Investigação Interdisciplinar em Sanidade Animal, Universidade de Lisboa, Lisbon, Portugal; ^2^Escola Superior de Tecnologia da Saúde de Lisboa, Instituto Politécnico de Lisboa, Lisbon, Portugal; ^3^Departamento de Biología Molecular, Facultad de Medicina, Universidad de Cantabria, Santander, Spain; ^4^Spanish National Cancer Research Center, CNIO, Madrid, Spain; ^5^Centro de Química Estrutural – Faculdade de Ciências da Universidade de Lisboa, Lisbon, Portugal

**Keywords:** colchicine, tubulin cofactors, TBCA, TBCB, TBCE, tubulin heterodimer dissociation, microtubule cytoskeleton

## Abstract

Colchicine has been used to treat gout and, more recently, to effectively prevent autoinflammatory diseases and both primary and recurrent episodes of pericarditis. The anti-inflammatory action of colchicine seems to result from irreversible inhibition of tubulin polymerization and microtubule (MT) assembly by binding to the tubulin heterodimer, avoiding the signal transduction required to the activation of the entire NLRP3 inflammasome. Emerging results show that the MT network is a potential regulator of cardiac mechanics. Here, we investigated how colchicine impacts in tubulin folding cofactors TBCA, TBCB, and TBCE activities. We show that TBCA is abundant in mouse heart insoluble protein extracts. Also, a decrease of the TBCA/β-tubulin complex followed by an increase of free TBCA is observed in human cells treated with colchicine. The presence of free TBCA is not observed in cells treated with other anti-mitotic agents such as nocodazole or cold shock, neither after translation inhibition by cycloheximide. *In vitro* assays show that colchicine inhibits tubulin heterodimer dissociation by TBCE/TBCB, probably by interfering with interactions of TBCE with tubulin dimers, leading to free TBCA. Manipulation of TBCA levels, either by RNAi or overexpression results in decreased levels of tubulin heterodimers. Together, these data strongly suggest that TBCA is mainly receiving β-tubulin from the dissociation of pre-existing heterodimers instead of newly synthesized tubulins. The TBCE/TBCB+TBCA system is crucial for controlling the critical concentration of free tubulin heterodimers and MT dynamics in the cells by recycling the tubulin heterodimers. It is conceivable that colchicine affects tubulin heterodimer recycling through the TBCE/TBCB+TBCA system producing the known benefits in the treatment of pericardium inflammation.

## Introduction

Microtubules (MTs) are dynamic polymers of heterodimers of α- and β-tubulin that grow and shrink by the addition and removal of tubulin heterodimers at their ends. MT arrays are present in almost all eukaryotic cells constituting, together with actin and intermediate filaments, the cytoskeleton. MTs, in crosstalk with other cytoskeleton members, play many roles in cell spatial organization, the establishment of cellular asymmetries and polarity, intracellular transport, cell migration, and morphogenesis (Dogterom and Koenderink, 2019). These polymers are also the main constituents of complex structures such as the mitotic and meiotic spindles, centrioles/basal bodies, and the ciliary axoneme, being involved in cell division, motility, and signaling. The diversity of MT functions depends on the biochemical variety of tubulin pools generated either by the constitutive or tissue-specific expression patterns of the tubulin gene family members and by tubulin post-translational modifications (Roll-Mecak, 2020). Additionally, MTs are modulated by a vast number of MT-binding proteins with multiple activities (for review, Goodson and Jonasson, 2018).

*In vivo*, the dynamic behavior of MTs relies on the existence of competent tubulin heterodimers originated either from *de novo* synthesis or MTs recycling (for review Gonçalves et al., 2010). For this, eukaryotic cells are equipped with a few molecular chaperones (cytosolic chaperonin CCT (cytosolic chaperonin-containing TCP1) and its cochaperone prefoldin) (Cowan and Lewis, 2001; Lopez et al., 2015), and a group of specific tubulin cofactors (TBCA-E) (Lewis et al., 1997; Lopez-Fanarraga et al., 2001). Along with tubulin folding, tubulin cofactors assist tubulin heterodimer assembly/dissociation, as well as tubulin degradation (Lewis et al., 1997; Lopez-Fanarraga et al., 2001; Kortazar et al., 2007; Voloshin et al., 2010). Therefore, *in vivo*, tubulin cofactors play pivotal roles in maintaining tubulin pools and tubulin heterodimers recycling/degradation by controlling their native structure’s quality. This also indicates that TBCs can be involved in MT cytoskeleton remodeling and the assembly/disassembly of specific MT populations and structures. The overexpression of TBCD and TBCE completely disrupts the MT cytoskeleton (Martín et al., 2000; Kortazar et al., 2006). TBCD and TBCE are capable of dissociating the tubulin heterodimer by themselves, but in the case of TBCE, its dissociation activity is highly increased by the presence of TBCB (Serna and Zabala, 2016). In fact, TBCB and TBCE stabilize the α-tubulin subunit after dimer dissociation originating a ternary complex TBCE/TBCB/α-tubulin (Carranza et al., 2013; Serna et al., 2015). Both TBCB and TBCE share a CAP-Gly and a UBL (ubiquitin-like) domain in their primary structures, but in inverted order (Serna and Zabala, 2016). The dissociation complex may also be involved in α-tubulin degradation. The UBL domains of TBCE and TBCB present a protruding arrangement suggesting the possibility of interacting with the proteasome (Serna et al., 2015), as mechanistically detailed in the review by Serna and Zabala (2016).

After tubulin heterodimer dissociation, cells avoid the toxic effect of β-tubulin (Archer et al., 1995; Abruzzi et al., 2002) by stabilizing it through the association with TBCD or/and TBCA (Carranza et al., 2013; Serna et al., 2015). In budding yeast, TBCA (Rbl2p) can rescue cells from β-tubulin overexpression and is required for meiosis (Archer et al., 1995). In contrast, in fission yeast, TBCA (Alp31) is necessary for the integrity of MTs and consequently for growth polarity (Radcliffe et al., 2000). *In vitro*, TBCA is dispensable for tubulin folding (Abruzzi et al., 2002), which contrasts with the observation that, in human cell lines, TBCA is encoded by an essential gene (Nolasco et al., 2005). Depletion of TBCA in human cells causes a decrease in soluble tubulin, modifications in MT and actin cytoskeletons organization, and G1 cell cycle arrest. Similarly, TBCA mutants in *Schizosaccharomyces pombe* and *Arabidopsis thaliana* present unstable and defective MTs structures, compromised MT organization, and cell morphology alterations (Archer et al., 1995; Radcliffe et al., 2000; Kirik et al., 2002; Steinborn et al., 2002).

Although the molecular mechanisms underlying the role of tubulin cofactors in tubulin heterodimers’ maturation, tubulin recycling, and tubulin degradation have been progressively elucidated, the complete scenario of their roles *in vivo* is far from being completely understood. Different tubulin cofactors seem to play critical roles in invertebrate and vertebrate brain function/maintenance, development, and morphogenesis (Shern et al., 2003; Okumura et al., 2015; Chen et al., 2016), which is highlighted by their association with diverse neurodegenerative diseases like the giant axonal neuropathy (GAN) (Wang et al., 2005; Yang et al., 2007), amyotrophic lateral sclerosis (Helferich et al., 2018), motor neuronopathy (Martin et al., 2002; Schaefer et al., 2007; Bellouze et al., 2014), progressive neurodegenerative encephalopathy with distal spinal muscular atrophy (Sferra et al., 2016) and eventually with tauopathies (Fujiwara et al., 2020). Other syndromes, such as the recessive disorder Sanjad-Sakati syndrome (SSS), and the autosomal recessive Kenny-Caffey syndrome, were associated with TBCE mutations (Parvari et al., 2002). In general, the cells from patients with these syndromes show lower MT density/disrupted MT networks, perturbed MT polarity, and Golgi apparatus fragmentation (Martin et al., 2002; Schaefer et al., 2007; Bellouze et al., 2014; Sferra et al., 2016). TBCs have also been involved in breast cancer development suggesting that dysregulation of the tubulin heterodimer pool may impact tumor cell phenotypes and response to chemotherapy (Vadlamudi et al., 2005; Hage-Sleiman et al., 2010). The activity of tubulin cofactors has also been associated with cilia biology (Grayson et al., 2002; Lopez-Fanarraga et al., 2007; Fanarraga et al., 2010a). The complexity of the roles of tubulin cofactors indicates that these proteins may also have non-tubulin-folding activities. For example, TBCB and TBCD localize at the centrosome, and changes in these cofactors affect centrosomal γ-tubulin (Vadlamudi et al., 2005; Cunningham and Kahn, 2008; Fanarraga et al., 2010a). In *S. pombe*, genetic interactions between TBCD mutant forms with the kinetochore CENP-B-like protein and spindle components (Fedyanina et al., 2006) suggest that TBCD may play essential functions at these structures. Also, disassembly of tight and adherent junctions followed by cell dissociation from the epithelial monolayer is observed in TBCD overexpression backgrounds (Shultz et al., 2008). The study of the complexity of regulatory mechanisms involving tubulin cofactors has been neglected. Still, the activity of TBCB is regulated by post-translational modifications like phosphorylation by a p21-activated kinase (Pak1) that is essential for the polymerization of new MTs (Vadlamudi et al., 2005). Similar to *tau* and α-tubulin, TBCB also undergoes nitration, which inhibits the polymerization of new MTs (Rayala et al., 2007) by inhibiting TBCB phosphorylation. TBCB also seems to be regulated by the non-coding micro RNA miR-1825 (Helferich et al., 2018), whereas a non-coding antisense RNA regulates TBCA during testis maturation (Nolasco et al., 2012).

MTs and/or tubulin are the targets for many small molecules that act as anti-mitotic agents. However, how these compounds interfere with the *in vivo* activities of TBCs is unknown. The importance of investigating this putative interplay relies not only on the fact that valuable information about the mechanisms underlying MT dynamics *in vivo* can be acquired but primarily because these molecules are clinically significant. For example, the anti-mitotic drug colchicine has been used as an anti-inflammatory and anti-fibrotic drug (for review, Roberts, 1987; Dalbeth et al., 2014). The uses for colchicine include chronic inflammatory diseases as gouty arthritis (Roberts, 1987; Dalbeth et al., 2014), the familial Mediterranean fever (FMF) (Goldfinger, 1972), the Behcet’s disease (Yurdakul et al., 2001), a variety of dermatological conditions (e.g., epidermolysis bullosa acquisita and aphthous stomatitis), and other fibro-inflammatory diseases (Addrizzo-Harris et al., 2002; Martínez et al., 2015; Solak et al., 2017). In the last years, colchicine has also been emerging as a first-line drug to treat pericardial diseases. The anti-inflammatory properties of colchicine and the absence of effective drugs to treat COVID-19 lead to the idea that the use of colchicine may be efficient in COVID-19 treatment (Corral et al., 2020; Nasiripour et al., 2020). Therefore, several colchicine randomized controlled trials in COVID-19 are ongoing^1^.

The anti-inflammatory action of colchicine seems to result from a compromised MT cytoskeleton that affects several inflammatory pathways in cells that mediate immune response like the adhesion and recruitment of neutrophils, superoxide production, inflammasome activation, the RhoA/Rho effector kinase (ROCK) pathway, and the tumor necrosis factor-alpha (TNF-α)-induced nuclear factor κB (NF-κB) pathway (for review Angelidis et al., 2018).

Virtually nothing is known about colchicine’s impact on the recycling cycle and quality control of tubulin heterodimers conducted by TBCs. Here we show that the exposure of human cells to colchicine causes a decrease of the TBCA/β-tubulin complex to vestigial levels followed by an increase of free TBCA. Free TBCA was never observed in human control cells nor in cells exposed to other anti-mitotic MT depolymerizing agents like nocodazole and cold-shock, or even after translation inhibition by cycloheximide. The appearance of free TBCA is accompanied by an increase in free soluble tubulin heterodimers due to MT depolymerization. We show, in *in vitro* assays, that colchicine inhibits tubulin heterodimer dissociation by TBCE/TBCB, affecting heterodimer recycling/quality control. This result strongly suggests that MT depolymerization by colchicine treatment is not only due to the conformational alteration of the tubulin heterodimer but also to the inability of cofactors to recycle these heterodimers, which also explains the irreversible colchicine action. The manipulation of TBCA levels, either by RNAi or by overexpression, causes the decrease of tubulin heterodimers. Collectively, our data strongly suggest that TBCA is mainly receiving β-tubulin from the dissociation of pre-existing heterodimers instead of newly synthesized tubulins, which is supported by the observations using the translation inhibitor cycloheximide. Thus, we show that the system TBCE/TBCB+TBCA is crucial for the control of the critical concentration of free tubulin heterodimers and MT dynamics in the cells. The finding that colchicine affects the tubulin heterodimer recycling/degradation system TBCE/TBCB+TBCA should be taken into account in the context of colchicine’s therapeutic benefits as an anti-inflammatory drug.

## Results

### Colchicine Causes the Decrease of the TBCA/β-Tubulin Complex and the Detection of Free TBCA in HeLa Cells

In addition to tubulin folding, TBCs are also involved in tubulin heterodimer recycling and degradation, regulating MT dynamics by controlling the tubulin heterodimer pool competent to polymerize (Serna et al., 2015). MTs and/or tubulin are targets for many small molecules that can stabilize or destabilize these polymers due to their ability to increase or decrease MT assembly at high concentrations. These molecules also strongly compromise MT dynamics at concentrations 10- to 100-fold lower than those required to affect MT mass (for review, see Dumontet and Jordan, 2010).

Previously, we showed that TBCA knockdown causes a decreased amount of α- and β-tubulin, G1 cell cycle arrest, and cell death in human cell lines (Nolasco et al., 2005). Contrary to TBCB, TBCE, and TBCD, TBCA is not able to dissociate the native tubulin heterodimer by itself (Martín et al., 2000; Kortazar et al., 2006, 2007; Carranza et al., 2013), but after tubulin heterodimer dissociation, TBCA forms a stable complex with β-tubulin, being responsible for its recycling/degradation (Kortazar et al., 2007).

**FIGURE 1:**
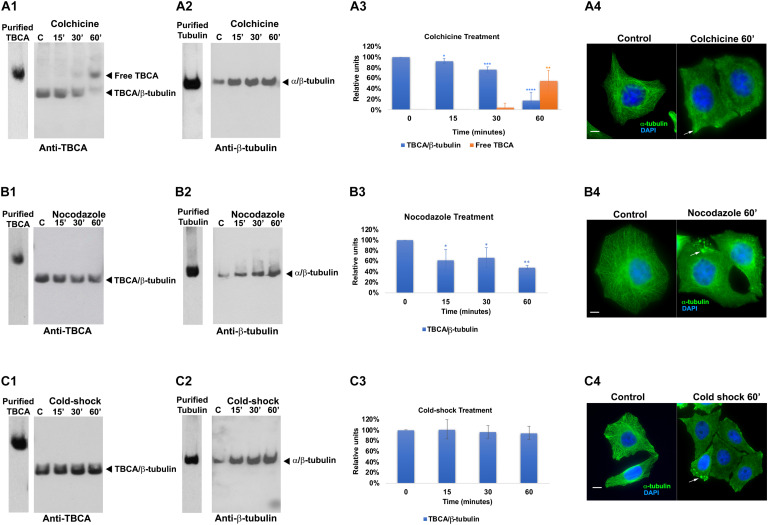
Colchicine treatment triggers the appearance of free TBCA in HeLa cells. HeLa cells were treated with different microtubule depolymerizing agents (colchicine, nocodazole, and cold shock) for 15, 30, and 60 min. After each treatment, native protein extracts were prepared, and equal amounts of soluble protein extracts (30 μg) were analyzed by non-denaturing gel electrophoresis (6% native-PAGE) followed by western blot with antibodies directed to TBCA **(A1,B1,C1)** and β-tubulin **(A2,B2,C2)**. Representative images of the native-PAGE show that: free TBCA was not detected in all-control cells (untreated) **(A1,B1,C1)**; upon colchicine treatment, the amount of TBCA/β-tubulin complex progressively decreases over the indicated time-frames reaching the lowest level at 60 min which is accompanied by the detection of free TBCA in the cell extract (**A1,A3**: *p < 0.05, **p < 0.01, ***p < 0.005, and ****p < 0.001); under nocodazole treatment TBCA/β-tubulin complex also decreases at 60 min to exposure of cells to this anti-mitotic drug, but free TBCA is never detected (**B1,B3**: *p < 0.05 and **p < 0.0001); and under cold shock treatment the levels of the TBCA/β-tubulin complex are maintained **(C1,C3)**. The soluble α/β-tubulin levels increase in response to MT depolymerization **(A2,B2,C2)**. The graphs in panels **(A3)** (colchicine), **(B3)** (nocodazole), and **(C3)** (cold-shock), show the relative units in percentage of TBCA/β-tubulin complex and free TBCA levels in comparison to control cells (100%). Statistical significance was calculated using a *t*-test and the *p*-values were determined in comparison to the time 0 min (control). The graphic bars are the mean s.d. (error bars) of three independent assays. For each treatment, the MT depolymerization was confirmed by indirect immunofluorescence using an antibody against α-tubulin **(A4,B4,C4)** (the DNA was stained with DAPI, scale bar 10 μm).

To investigate how the *in vivo* activities of TBCA are affected by MT depolymerizing agents, we treated HeLa cells with: a) colchicine (5 μM), a tubulin-binding anti-mitotic drug, with clinical relevance, that depolymerizes MTs irreversibly; b) nocodazole (30 μM), a tubulin-binding anti-mitotic drug, a rapid and reversible inhibitor of MT polymerization (De Brabander et al., 1976); and c) cold shock, low temperature, that is well-established to promote MT depolymerization (Weisenberg, 1972; Shelanski et al., 1973). The exposure of cells to all different treatments was performed for 15, 30, and 60 min after which soluble native protein extracts were prepared and analyzed by 6% (w/v) non-denaturing-PAGE. As migration markers, purified α/β-tubulin heterodimers and TBCA were simultaneously analyzed with the protein extracts of cells treated with distinct MT depolymerizing agents (Figure 1). These analyses were followed by western blot using antibodies against TBCA and β-tubulin (Figure 1). As expected for purified TBCA (free TBCA), we observed a unique band in the lane (Figures 1A1,B1,C1). However, according to Llosa et al. (1996) when protein extracts from cells are analyzed in this type of native gels, it is expected that the anti-TBCA sera will be able to detect not only the free TBCA but also a faster migrating band corresponding to the TBCA/β-tubulin complex.

Interestingly, in control extracts from HeLa cells (Figures 1A1,B1,C1; lane C), the band corresponding to free TBCA is never observed. A faster migrating band, probably corresponding to the TBCA/β-tubulin complex, is clearly visible. To demonstrate that in our analysis, we could not use the antibody against β-tubulin as this only detects the α/β-tubulin heterodimer but not the TBCA/β-tubulin complex (Figures 1A2,B2,C2). This is probably a consequence of the β-tubulin epitope, recognized by this antibody, being hidden in the quaternary structure of the TBCA/β-tubulin complex. Therefore, to prove that the faster migrating band corresponds to the TBCA/β-tubulin complex, we excised this band from a 6% (w/v) Native-PAGE stained with Coomassie Brilliant Blue and re-analyzed it on a 16.5% (w/v) Tricine–SDS–PAGE. Then western-blot analysis was performed using specific antibodies to TBCA and β-tubulin. This analysis showed that the extracted band possesses β-tubulin and TBCA, confirming that it corresponds to the TBCA/β-tubulin complex (Supplementary Figure 1).

Curiously, these results clearly show that, in HeLa control cells, most TBCA is in a complex with β-tubulin, and these cells do not seem to contain a free pool of TBCA. The results in HeLa cells exposed for different times to cold shock 4°C (Figure 1C1) are like those observed in control cells, and the amount of the TBCA/β-tubulin complex does not significantly change over the studied time lapse (see panel C3). On the contrary, the amount of soluble tubulin heterodimer increases reaching the highest level at 60 min after exposure to 4°C (Figure 1C2), suggesting that depolymerized MTs contribute to the rise of the soluble tubulin pool (heterodimers). In fact, the detection of α-tubulin by immunofluorescence (IF) microscopy in HeLa control cells and cells exposed to cold shock for 60 min show that in cells subject to 4°C the MT arrays are dismantled, even though their regular shape is maintained (Figure 1C4). In some cells, soluble free tubulin concentrates at specific sites of the cytoplasm (Figure 1C4 arrows). Noteworthy, depolymerization of MTs caused by nocodazole or colchicine as revealed by IF microscopy analysis (see Figures 1A4,B4) is accompanied by the decrease of the amount of the TBCA/β-tubulin complex (Figures 1A1,B1). However, this decrease is much more accentuated in HeLa cells subjected to colchicine treatment (18 ± 16%) than in those subjected to nocodazole (48 ± 4%) for the same period (Figures 1A3,B3).

Moreover, the reduction of the amount of TBCA/β-tubulin complex initiates at 15 min of colchicine and nocodazole treatment (Figures 1A1,A3). Strikingly, in cells treated with colchicine, the decrease of the amount of TBCA/β-tubulin complex is closely followed by the gradual appearance of a band corresponding to free TBCA (Figures 1A1,A3). This band is not observed in HeLa cells treated with nocodazole, indicating a specific effect of the colchicine action in HeLa cells. Moreover, in agreement with the IF microscopy data, a progressive increase in the amount of α/β-tubulin heterodimer is observed either in nocodazole or colchicine HeLa treated cells during the studied time course (see Figures 1A3,B3). Thus, the interesting observation that colchicine dramatically increases the levels of free TBCA is not a direct consequence of MT depolymerization.

For all conditions the soluble protein extracts previously tested in native gels were subjected to 16.5% (w/v) Tricine–SDS–PAGE followed by detection with anti-TBCA and β-tubulin antibodies upon western blot analysis. The results (Supplementary Figure 2) show no significant alterations of TBCA levels and a slight increase in β-tubulin levels compared to the levels of these proteins in control cells. Of note, the increase of α/β-tubulin heterodimers, in response to the MT depolymerization, is not as clearly observed as in native gels, since in these we analyze the amount of all β-tubulin polypeptides regardless of its origin (α/β-tubulin, TBCA/β-tubulin or other). Therefore, the variations in the amount of free TBCA and the TBCA/β-tubulin complex observed in colchicine and nocodazole treated cells (Figures 1A1,B1) do not correspond to changes in the total amount of soluble TBCA and β-tubulin proteins in HeLa cells treated with these different MT depolymerizing agents in the indicated time-frames. These data clearly show that the decreased amounts of the TBCA/β-tubulin complex are not the result of reduced levels of any of the two proteins in HeLa cells. Moreover, in the time course of all the conditions studied, the soluble β-tubulin levels do not change significantly (see Supplementary Figure 2), but the native analysis indicates that the soluble α/β-tubulin heterodimer amount clearly increases (see Figures 1A2,B2,C2). This strongly suggests that at least β-tubulin may be displaced to a complex not visible in our conditions.

### Free TBCA Is Not a Consequence of Protein Translation Inhibition

**FIGURE 2:**
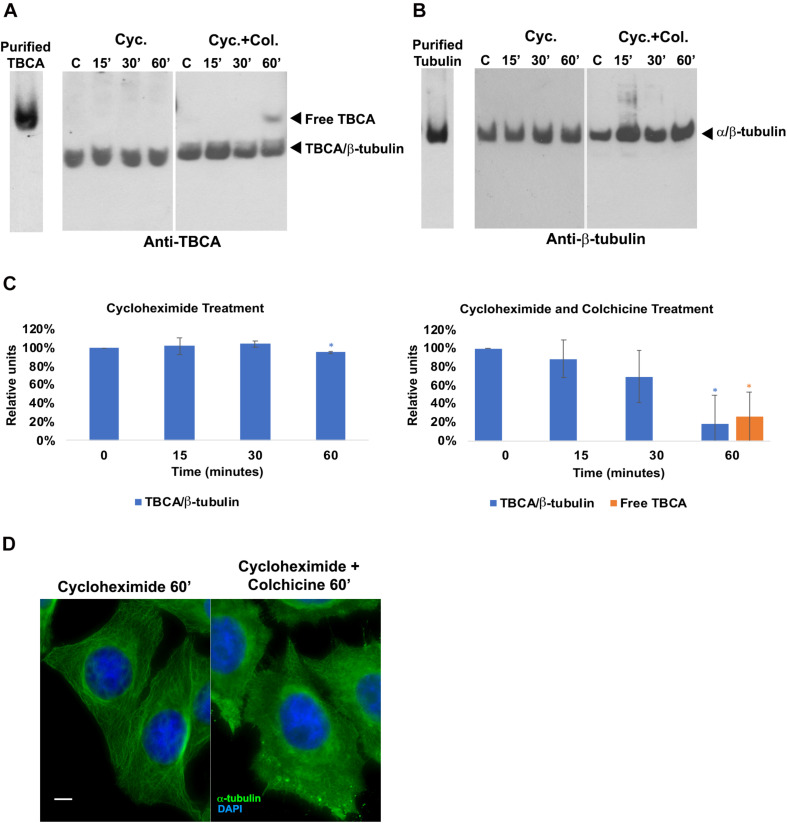
Protein translation inhibition by cycloheximide does not lead to the detection of free TBCA in the cell extract. HeLa cells were treated with cycloheximide to inhibit the protein translation and also with cycloheximide plus colchicine for 15, 30, and 60 min. In both treatments, time 0 min (control) corresponds to 15 min after the cycloheximide addition. After each treatment, native protein extracts were prepared, and equal amounts of soluble protein (30 μg) were analyzed by non-denaturing gel electrophoresis (6% Native-PAGE) followed by western blot with antibodies directed to TBCA **(A)** and β-tubulin **(B)**. Representative images of the native-PAGE show that under the cycloheximide treatment, free TBCA was not detected, and the levels of TBCA/β-tubulin are maintained but, the addition of colchicine to cycloheximide leads to a decrease in TBCA/β-tubulin levels and detection of free TBCA after 60 min of colchicine treatment (**A,C**: for cycloheximide treatment *p < 0.005, and for cycloheximide and colchicine treatment *p < 0.002). The graphs in panel **(C)** (cycloheximide and cycloheximide plus colchicine), show the relative units in percentage of TBCA/β-tubulin complex and free TBCA levels in comparison to control (100%) cells. Statistical significance was calculated using a *t*-test. And the *p*-values were determined in comparison to the time 0 min (control). The graphic bars are the mean s.d. (error bars) of three independent assays. The α/β-tubulin levels are maintained during cycloheximide treatment but increase after addition of colchicine **(B)**. For each treatment, the MT depolymerization was confirmed by indirect immunofluorescence **(D)** using an antibody against α-tubulin (the DNA was stained with DAPI, scale bar 10 μm). The results observed in panel **(B)** agree with the results showed in panel **(C)**, where MTs can be detected with cycloheximide treatment but not with cycloheximide plus colchicine **(C)**.

Since there is evidence that low doses of colchicine prevent mRNA translation by promoting polysome disaggregation (Walker and Whitfield, 1984), we put forward the hypothesis that the appearance of free TBCA in cells treated with colchicine could be related to the absence of newly synthesized tubulin. To assess this hypothesis, we analyzed the effect of the eukaryotic translational elongation inhibitor cycloheximide in the amounts of TBCA/β-tubulin complex as well as free TBCA. Thus, HeLa cells were treated with cycloheximide (50 μg/ml) for 15 min, 30 min, and 60 min, and soluble protein extracts were analyzed by 6% native-PAGE. We expected that the absence of input of newly synthesized tubulin to TBCA, due to protein synthesis inhibition, would mimic the effect of colchicine. However, we did not observe a decrease in the TBCA/β-tubulin complex levels nor in the free TBCA protein’s appearance during the time course of cycloheximide treatment (Figure 2C). So, translation blockage does not affect TBCA/β-tubulin complex levels and does not cause the appearance of free TBCA. These results indicate that the decrease in the amount of TBCA/β-tubulin in HeLa cells treated with colchicine or nocodazole is not a consequence of the inhibition of protein translation caused by the increase in soluble tubulin as a consequence of the loss of the MTs network. Also, the detection of free TBCA in cells treated with colchicine cannot be ascribed to the absence of newly synthesized β-tubulin. Thus, to further unequivocally show that the appearance of the free TBCA protein observed is essentially the result of colchicine treatment, we analyzed the soluble protein extracts of HeLa cells simultaneously treated with cycloheximide and colchicine. For this, we treated HeLa cells with cycloheximide (50 μg/ml) for 15 min before colchicine was added. This period provided the time to inhibit protein synthesis prior to the action of colchicine. From these cells, soluble protein extracts were prepared after 15, 30, and 60 min of exposure to both compounds and analyzed in a 6% native-PAGE followed by western blot analysis using specific antibodies against TBCA and β-tubulin (Figure 2). The pattern of variation of TBCA/β-tubulin complex levels and free TBCA protein appearance were like those obtained in cells only treated with colchicine (compare Figures 1A1, 2A,C). Therefore, colchicine causes the appearance of free TBCA. As anticipated, protein synthesis inhibition does not interfere with colchicine’s ability to promote MT depolymerization, which was confirmed by indirect IF microscopy (Figure 2D). All samples analyzed by non-denaturing gel electrophoresis (6% native-PAGE) were simultaneously analyzed by 16.5% (w/v) Tricine–SDS–PAGE followed by western blot analysis with antibodies directed to TBCA and β-tubulin. As observed in Supplementary Figure 2, colchicine in the presence of the translation inhibitor does not cause dramatic changes in the levels of soluble TBCA and β-tubulin (Supplementary Figure 3).

Overall, these results show that the absence of protein synthesis and consequently the absence of an input of newly synthesized β-tubulin in the tubulin folding pathway does not lead to the appearance of free TBCA nor affects the levels of the TBCA/β-tubulin complex. This is the first indication that TBCA is involved in heterodimer recycling from MT depolymerization. This observation may finally elucidate the apparent incongruent observations that TBCA is not essential for tubulin folding *in vitro* (Tian et al., 1996) while its depletion causes cell death (Nolasco et al., 2005).

### TBCB and TBCE Are Not Able to Dissociate Tubulin Heterodimers Bound to Colchicine

**FIGURE 3:**
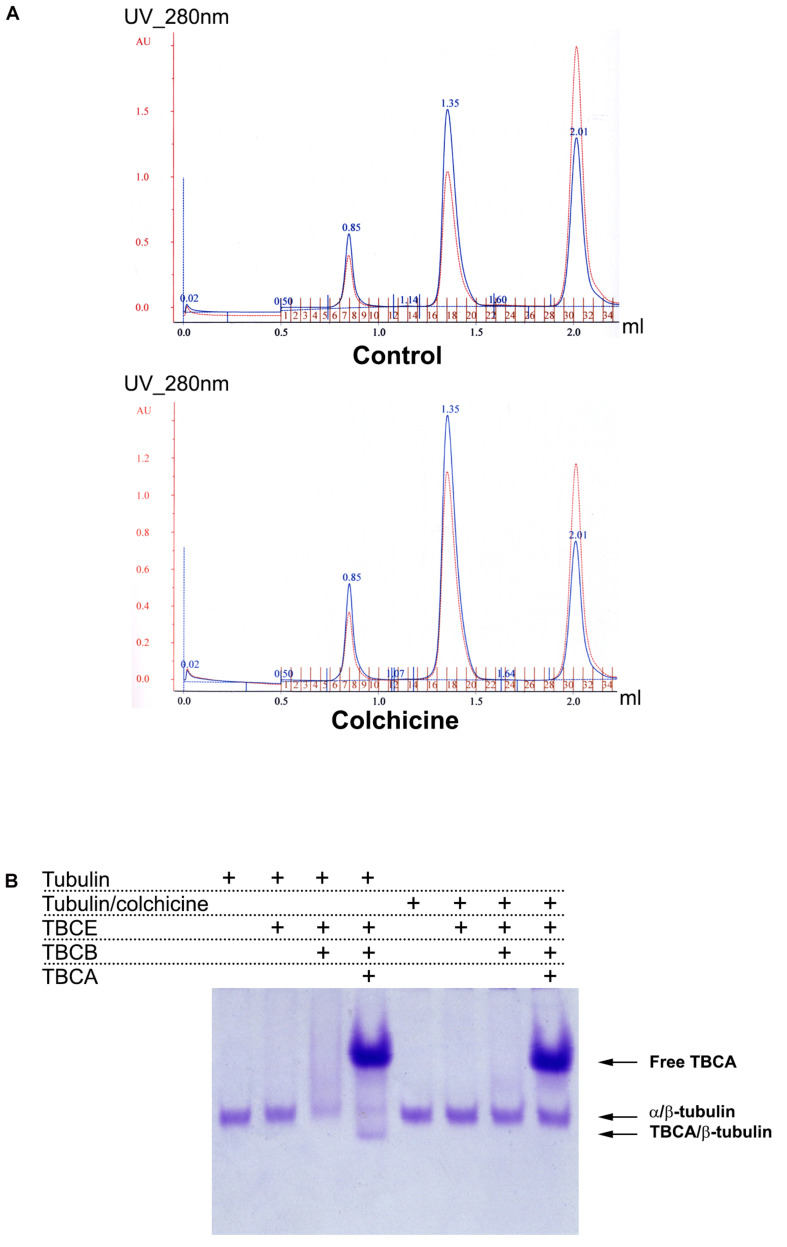
*In vitro* dissociation assays reveal that TBCB and TBCE are not able to dissociate the tubulin-colchicine heterodimer which leaves TBCA free. **(A)** After the assay of colchicine binding to tubulin, tubulin-colchicine heterodimers were purified by gel filtration to remove the unbounded colchicine molecules and aggregated misfolded tubulin. As a control the same amount of tubulin was also incubated without colchicine and purified by gel filtration. Chromatographic elution profiles obtained at 280 nm of absorbance from a gel filtration analysis of control and tubulin heterodimers incubated with colchicine are very similar, presenting three prominent peaks: the first one corresponds to tubulin aggregates, the second one to tubulin heterodimers, and the third one to small molecules. The colchicine molecule bound to tubulin heterodimer did not affect the behavior of tubulin heterodimer in gel filtration. Each peak of tubulin and tubulin-colchicine heterodimers were pooled (17–19 fractions). **(B)** Purified tubulin and tubulin-colchicine heterodimers, from pooled fractions (2.2 μg/reaction), were incubated at 30°C for 30 min with or without TBCE (1.5 μg/reaction), TBCB (0.6 μg/reaction) and TBCA (5 μg/reaction), according to the table, to perform tubulin heterodimer dissociation assays. The tubulin heterodimer dissociation assays were analyzed by non-denaturing gel electrophoresis (6% native-PAGE) and stained with Coomassie brilliant blue. The tubulin incubation with TBCE and/or TBCB lead to a decrease in the tubulin heterodimers amount due to tubulin heterodimer dissociation, as previously described (Kortazar et al., 2006; Carranza et al., 2013). When TBCA is also present, we detected a new band in the gel that corresponds to TBCA/β-tubulin (Kortazar et al., 2006; Carranza et al., 2013). When colchicine is bound to tubulin, TBCE and/or TBCB cannot dissociate the tubulin heterodimer. Consequently, in the presence of TBCA, we did not detect the presence of TBCA/β-tubulin complex. Colchicine impairs the tubulin dissociate activity of TBCE/TBCB, leaving TBCA free.

Next, we aimed at elucidating the intriguing observation that 60 min of colchicine treatment leads to the appearance of free TBCA. It is well-established that *in vitro*, TBCA is able to accept β-tubulin from native tubulin heterodimers in the presence of TBCE and TBCB (Kortazar et al., 2006, 2007). Additionally, TBCB and TBCE cooperate to dissociate the tubulin dimer and stabilize α-tubulin (Kortazar et al., 2007). Also, colchicine binds irreversibly to tubulin dimers (Bhattacharyya et al., 2008; Bhattacharya et al., 2016). Together, these data strongly suggest that TBCA cannot interact with β-tubulin from colchicine-bound to tubulin heterodimers, probably because colchicine affects the ability of TBCB/TBCE to dissociate the heterodimer. To test this hypothesis, we performed *in vitro* tubulin dissociation assays in the presence of purified tubulin cofactors: TBCE, TBCE plus TBCB, and TBCE plus TBCB plus TBCA. In these assays, we incubated these cofactors with native purified tubulin or with native tubulin purified after colchicine treatment. In the last case, tubulin was incubated with colchicine for 1 h at room temperature in a proportion of 2 μM:48.2 μM, respectively (Banerjee and Luduena, 1992). After incubating with colchicine, we purified the tubulin heterodimer-colchicine complex by gel filtration chromatography to remove the unbound colchicine. A similar purification protocol was applied to tubulin not incubated with colchicine (control). Figure 3A shows the chromatograms of the tubulin and tubulin-colchicine gel filtration purification. The separation profiles are very similar, indicating that colchicine did not affect tubulin heterodimer behavior through gel filtration chromatographic analysis. The tubulin heterodimer elutes at the second peak (fractions 17–19) in accordance with its expected size (Fanarraga et al., 2010b). Tubulin also appears in the column’s void volume (first peak; fractions 6–9), suggesting the formation of large aggregates (Fanarraga et al., 2010b). In each case, fractions corresponding to tubulin heterodimers peak (17–19 fractions in both cases) were pooled. These fractions were used to perform tubulin heterodimer dissociation assays (Kortazar et al., 2006). For this purified tubulin and tubulin-colchicine heterodimers (2.2 μg/reaction) were incubated with or without TBCE (1.5 μg/reaction), TBCB (0.6 μg/reaction) and TBCA (5 μg/reaction), according to the table in Figure 3B. The products of tubulin heterodimer dissociation assays were analyzed by native-PAGE, and the gel was stained with Coomassie brilliant blue. Under these conditions, tubulin incubation with TBCE and TBCB leads to decreased tubulin heterodimers’ concentration due to tubulin heterodimer dissociation, which agrees with what was previously described by Kortazar et al. (2006). As expected (Kortazar et al., 2006; Carranza et al., 2013), when TBCA is included in the reaction, a new band corresponding to TBCA/β-tubulin complex is detected in the gel. However, when a similar assay is performed using tubulin pre-incubated with colchicine, the decrease in the α/β-tubulin heterodimer is not observed (Figure 3B). Consequently, in the presence of TBCA, we did not detect the TBCA/β-tubulin complex. These results clearly indicate that the binding of colchicine to tubulin heterodimer blocks the ability of TBCE and TBCB to dissociate the tubulin heterodimer, which leaves TBCA free.

**FIGURE 4:**
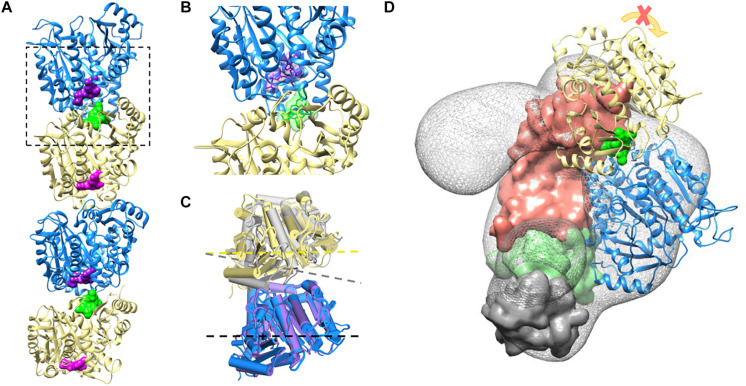
Colchicine could impair TBCE-TBCB complex function in the α/β-tubulin heterodimer dissociation process by stabilizing the α-tubulin/β-tubulin interface. **(A)** Two associated α/β-tubulin heterodimers stabilized by the colchicine molecule (green surface). The GTP form of the α-tubulin monomer and the GDP form of the β-tubulin are displayed in blue and yellow (GTP and GDP surfaces are shown in purple and magenta, respectively), PDB ID 4O2B. **(B)** Zoom-in view of colchicine (green) at the α/β-tubulin interface, next to the α-tubulin GTP binding pocket, PDB ID 4O2B (Prota et al., 2014). **(C)** Comparison of the free α/β-tubulin heterodimer (α-tubulin in purple and β-tubulin in gray, PDB ID 1JFF; Löwe et al., 2001), with the α/β-tubulin heterodimer stabilized by colchicine (α-tubulin in blue and β-tubulin in yellow, PDB ID 4O2B; Prota et al., 2014) by aligning the α-tubulin monomers. The presence of colchicine affects the α/β-tubulin interface geometry, as shown with the dash lines. **(D)** The complex TBCE-TBCB, hold by the interaction between the CAP-Gly domains (TBCE CAP-Gly domain surface in green and TBCB CAP-Gly domain surface in gray), binds the α-tubulin monomer of the α/β-tubulin heterodimer (blue, EMDB-2447; Serna et al., 2015) pushing the TBCE LRR domain (domain surface in coral) toward the β-tubulin monomer (yellow) and distorting the α/β-tubulin interface. The presence of colchicine (green surface) could impair α/β-tubulin heterodimer dissociation by stabilizing the α/β-tubulin interface, making it resistant to the mechanical force applied by the LRR domain.

These data are supported by a structural prediction based on the comparison between the α/β-tubulin-colchicine and α/β-tubulin 3D structure in association with TBCB and TBCE as presented in Figure 4. This analysis was based on the fact that the tubulin dimer dissociation by TBCB/TBCE involves a ternary complex TBCE/TBCB/α-tubulin (Carranza et al., 2013; Serna et al., 2015). TBCE and TBCB have a cytoskeleton-associated protein glycine-rich (CAP-Gly) and a UBL domain (Grynberg et al., 2003; Serna and Zabala, 2016). The TBCB intermediate region has a short coiled-coil region (CC), whereas that of TBCE has a leucine-rich repeat (LRR) domain. The molecular architecture of this complex proposed by Serna et al. (2015), predicts that the TBCE-TBCB complex, hold by the interaction between the CAP-Gly domains, bind the α-tubulin monomer of the α/β-tubulin heterodimer and dissociate it presumably by pushing the TBCE LRR domain toward the β-tubulin monomer and distorting the α/β-tubulin interface. Also, the colchicine molecule binds at the α/β-tubulin interface, next to the α-tubulin GTP binding pocket (Prota et al., 2014). The comparative analysis of α/β-tubulin-colchicine and α/β-tubulin 3D structures show that, in the presence of the colchicine, the α/β-tubulin interface adopts a closer conformation (Figure 4C). This agrees with the experimental observations that colchicine binding induces a conformational change of tubulin (Garland, 1978; Detrich et al., 1981; Andreu and Timasheff, 1982). Therefore, the presence of the colchicine could impair the α/β-tubulin heterodimer dissociation by TBCB/TBCE by stabilizing the α/β-tubulin interface and make it resist the mechanical force applied by the LRR domain of TBCE (Figure 4D).

Again, the overall data strongly supports that an important role of TBCA in HeLa cells is to accept β-tubulin resulting from α/β-tubulin heterodimer that arises from MT depolymerization and is dissociated by the TBCB/TBCE machine or by the TBCD cofactor (Martín et al., 2000). Therefore, *in vivo*, TBCA’s role is articulated with TBCB/TBCE activity to recycle the tubulin heterodimers and most probably to control their quality. The three tubulin cofactors also probably manage tubulin degradation, with TBCA avoiding the toxic effect of β-tubulin during the recycling process. These data also explain why the depolymerization of MTs by colchicine is irreversible in the presence of saturated doses of this molecule. Tubulin-colchicine heterodimers are condemned to be part of a pool of heterodimers that cannot polymerize because they cannot be dissociated and then recycled.

### Studies of Overexpression and Depletion Support TBCA Main Role in β-Tubulin Recycling

**FIGURE 5:**
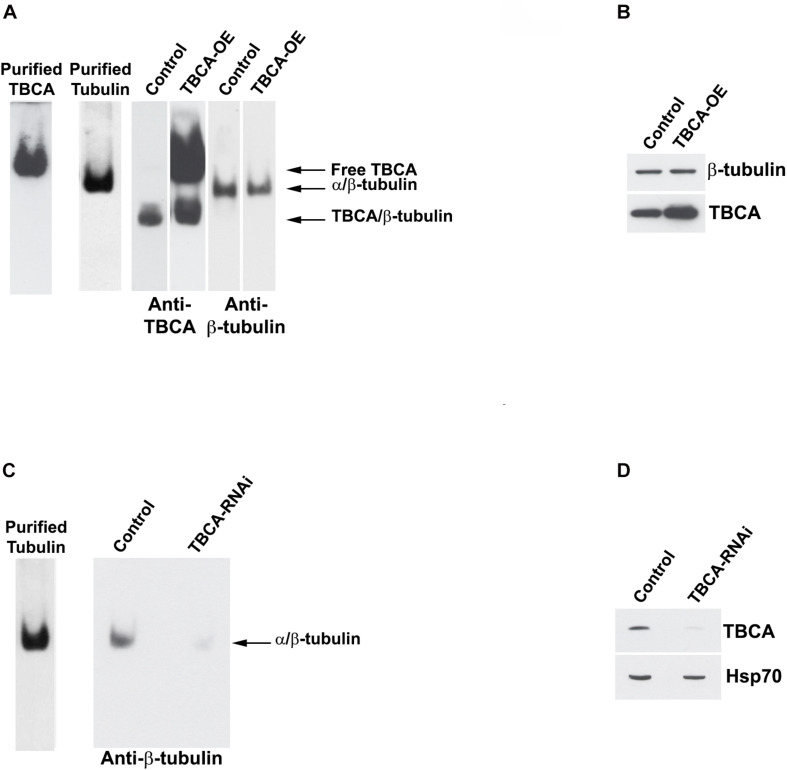
Native tubulin heterodimers levels decrease in response to TBCA overexpression and TBCA siRNA. HeLa cells were transfected with TBCA-pcDNA3 (TBCA-OE), and control cells were transfected with empty pcDNA3. 24 h after transfection, native protein extracts were prepared. Equivalent amounts of protein soluble fraction (30 μg) were analyzed by non-denaturing gel electrophoresis Native-PAGE) **(A)** and by 16.5% Tricine–SDS–PAGE **(B)** followed by Western blot with antibodies directed to TBCA and β-tubulin. **(A)** In native gels, TBCA overexpression is clearly detected by the increase of the amount of free TBCA and the TBCA/β-tubulin complex. Tubulin heterodimer levels decrease when TBCA is overexpressed. **(B)** The denaturing analysis using Tricine–SDS–PAGE showed that β-tubulin levels do not change in response to TBCA overexpression. 24 h after TBCA siRNA transfection (see Nolasco et al., 2005 for details and controls), protein extracts were prepared. Equal amounts of protein soluble fraction (30 μg) were analyzed by non-denaturing gel electrophoresis (6% Native-PAGE) **(C)** and by 16.5% Tricine–SDS–PAGE **(D)**, to check the TBCA knockdown efficiency, followed by western blot with antibodies against β-tubulin, TBCA and Hsp70. **(C)** TBCA knockdown also causes a decrease in the tubulin heterodimer amount. **(D)** Cells transfected for 24 h with TBCA siRNAs show a reduction in levels of TBCA. Hsp70 protein levels were used as a loading control.

The observation that an important role of TBCA is to bind β-tubulin during tubulin heterodimer recycling raised the question of whether TBCA levels may affect the tubulin dissociation rate by TBCB/TBCE, regulating, therefore, the availability of tubulin heterodimers competent to polymerize and also MT dynamics. It is conceivable, therefore, that if *in vivo* TBCA concentration is critical, it would regulate the efficiency of tubulin heterodimer dissociation by TBCB/TBCE. On other words, low levels of TBCA in the context of accentuated MT depolymerization could impair tubulin heterodimer dissociation by TBCB/TBCE since β-tubulin cannot be accepted, becoming toxic. This would implicate that in this background β-tubulin is driven to degradation. Consequently, we performed experiments where we modulated TBCA levels either by promoting its overexpression via pcDNA3-TBCA transfection (Figures 5A,B) or by knocking down the TBCA gene with a specific siRNA (as in Nolasco et al., 2005; Figures 5C,D). Western blot analysis of TBCA levels in control and TBCA siRNA-treated cells shows that TBCA levels decreased by about 48 ± 8% in TBCA-silenced cells. Hsp70 was used as loading control (Figure 5D). 24 h after transfections, native soluble protein extracts were prepared from control and TBCA overexpressing or depleted cells and analyzed either by native-PAGE or Tricine–SDS–PAGE followed by western blot using antibodies against TBCA, α- and β-tubulin (Figure 5). TBCA overexpression results show a dramatic increase in free TBCA and TBCA/β-tubulin complex levels in native gels (Figure 5A) and a clear increase in total soluble TBCA amount can be observed in SDS denaturing gels (Figure 5B).

On the other hand, the levels of α/β-tubulin heterodimer slightly decrease in response to the increased TBCA levels (Figure 5A). This small decrease in heterodimer may result from an increased dissociation rate of the heterodimer by TBCB/TBCE, which agrees with the increased levels of TBCA/β-tubulin complex (see Figure 5A) and absence of variation of total soluble β-tubulin levels. However, the results did not support the observation that TBCA improves the *in vitro* dimerization rate of α/β-tubulin (Fanarraga et al., 1999) since we do not observe an increase in tubulin heterodimer. This observation confirms once more that TBCA plays a critical role in heterodimer recycling but not in the tubulin folding route. On the other hand, TBCA siRNA significantly decreases the amount of native α/β-tubulin heterodimer (Figure 5C) in line with our previous results showing an accentuated decrease of α- and β-tubulin in HeLa and MCF-7 cell lines extracts in response to TBCA depletion (Nolasco et al., 2005). Probably this is due to the continuous tubulin heterodimer dissociation by TBCB/TBCE to be recycled. Since there is not enough TBCA to accept β-tubulin, both tubulins are probably destined to degradation. Altogether, these results indicate that the levels of TBCA are essential to regulate the rate of tubulin heterodimer dissociation/recycling by TBCB/TBCE. These results also strengthen the role of TBCA as a major component of the recycling/degradation pathway of the α/β-tubulin heterodimer, weakening its eventual role in the folding of newly synthesized β-tubulin *in vivo*.

### TBCA Is Abundant in Insoluble Protein Extracts of the Heart

**FIGURE 6:**
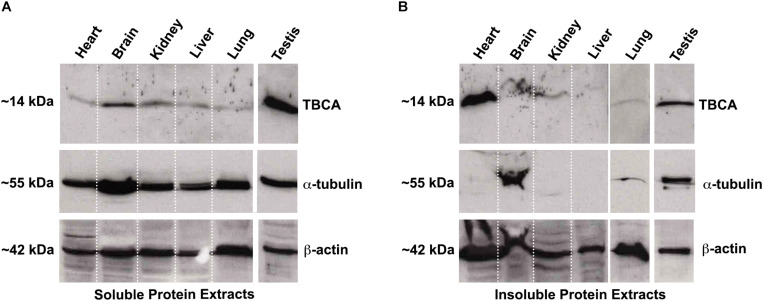
TBCA is present in a high amount in mouse insoluble protein extracts from the heart. Soluble **(A)** and insoluble **(B)** protein extracts from mouse organs (heart, brain, kidney, liver, lung, and testis) were prepared and analyzed on 16.5% (w/v) Tricine–SDS–PAGE followed by western blot with antibodies against TBCA, α-tubulin, and β-actin. Soluble protein extracts were quantified, and equal protein amounts (100 μg) were analyzed. Insoluble protein extracts were solubilized with 8 M urea and diluted 1:2 in loading buffer and, 20 out of 200 μl were analyzed. **(A)** The analysis showed that TBCA was more abundant in testis and brain. α-tubulin and β-actin are detected in all organs. **(B)** The insoluble protein extracts show that TBCA is more abundant in the heart and testis. α-tubulin is mainly detected in testis and brain, and β-actin is detected in all organs. The approximate molecular mass of the proteins is indicated on the left side of the panels.

Colchicine has been consistently used in the treatment of acute and recurrent pericarditis (Adler et al., 2015). Moreover, other potential therapeutic uses for colchicine are the prevention of Atrial Fibrillation (AF) reappearance after cardiac surgery or AF ablation (Calkins et al., 2017), as well as in patients with coronary artery disease (Nidorf et al., 2014, 2020; Deftereos et al., 2015) and chronic heart failure. In this view and guided by our results showing that: (i) colchicine impairs tubulin heterodimer dissociation by TBCB/TBCE, leaving TBCA free of β-tubulin; (ii) TBCA has a regulatory role in tubulin heterodimer recycling and (iii) altered levels of TBCA have been associated with human diseases (see Table 1), we analyzed the pattern of TBCA expression in different mouse organs. Previous analyses showed that TBCA is a soluble protein highly expressed in testis and in brain (Fanarraga et al., 1999) but no information is available regarding expression of TBCA in the heart. Therefore, we have prepared total protein extracts from the heart, brain, kidney, liver, lung, and testis of adult mice. The extracts were fractionated in soluble and insoluble fractions and analyzed by 16.5% (w/v) Tricine–SDS–PAGE followed by western blot using specific antibodies against TBCA, α-tubulin, and β-actin (Figure 6). Similar to what was observed by Fanarraga et al. (1999), TBCA is extremely abundant in the soluble fraction of testis followed by the brain, and α-tubulin and β-actin are detected in all tissue-specific samples (Figure 6A). However, in the insoluble fraction, TBCA is extremely abundant in the heart, followed by the testis. As expected, α-tubulin is mainly detected in the brain and testis, and β-actin is present in all samples but much more abundant in the heart, followed by the lung. These results are surprising because TBCA is assumed to be a soluble protein pinpointing for a possible contamination of the insoluble fraction with soluble proteins. This hypothesis is, however, discarded by the observation that in the heart TBCA is barely detected in the soluble fraction. We could still consider that our protein extraction was inefficient in heart cells lysates, explaining the low levels of TBCA observed in the soluble fraction and its retention in the insoluble extract. However, the detection of α-tubulin and even actin excludes this hypothesis (Figures 6A,B). Therefore, TBCA should be associated with membranes and/or cellular organelles present in insoluble extracts.

**TABLE 1:** Altered TBCA expression in several diseases.

**Disease**	**Organ**	**TBCA expression**	**Molecule**	**Reference**
Cancer	Kidney	Overexpression	Protein	Zhang et al., 2013
	Skin (cell culture)	Overexpression (SIRT knock-down)	Protein	Wilking-Busch et al., 2018
	Breast	Overexpression	Protein	Fonseca-Sánchez et al., 2012
	Stomach	Overexpression	mRNA	Malta-Vacas et al., 2009
Alzheimer	Brain	Underexpression	Protein	Zhang et al., 2018
Parkinson	Brain	Overexpression	Protein	Werner et al., 2008
Osteoporosis	Bone	Underexpression	mRNA	Xia et al., 2017
Ankylosing spondylitis	Blood	Hypomethylation	DNA	Coit et al., 2019

## Discussion

The dynamic behavior of MTs is critical for the myriad functions these polymers play in eukaryotic cells. MT functions require a pool of tubulin heterodimers competent to polymerize from newly synthesized tubulins and from recycling tubulin heterodimers from pre-existing MTs. These processes are complex and assisted by a dedicated set of tubulin cofactors. Colchicine is a natural anti-mitotic agent with clinical relevance in treating inflammatory diseases that is known to irreversibly affect MT polymerization and dynamics. Although anti-mitotic agents’ action in MT assembly is well-established, their impact on tubulin heterodimers’ recycling pathway is mainly unknown.

In the present work, we report that colchicine treatment of HeLa cells, but not cold-shock or nocodazole, causes the appearance of free TBCA in protein extracts (Figure 1). This is an unexpected observation since, in all situations that we have studied, we have always found TBCA in a complex with β-tubulin. Moreover, this was accompanied by a marked decrease of the TBCA/β-tubulin complex. Our data also shows that the appearance of free TBCA cannot be ascribed to the interruption of the tubulin folding pathway. Indeed, protein translation inhibition by cycloheximide does not lead to free TBCA, and colchicine is still able to cause the appearance of free TBCA in its presence (Figure 2). These assays clearly show that not only colchicine interferes with TBCA’s role, but TBCA seems to receive β-tubulin polypeptides mostly from the tubulin heterodimer recycling pathway. The current model of α/β-tubulin heterodimer assembly (Lopez-Fanarraga et al., 2001; Serna and Zabala, 2016) predicts that, if the input of newly synthesized β-tubulin is inhibited by the absence of translation, TBCA becomes free, at least partially. In the recycling pathway of the α/β-tubulin heterodimers, their dissociation by TBCE and TBCB leads to a ternary complex of TBCB/TBCE/α-tubulin, whereas β-tubulin is stabilized by TBCD or TBCA (Carranza et al., 2013; Serna et al., 2015). Noteworthy, *in vitro*, the TBCA/β-tubulin complex is stable and easy to be detected (Kortazar et al., 2006). Therefore, the appearance of free TBCA is probably the result of compromised TBCB/TBCE dissociation activity due to colchicine. Our tubulin heterodimer dissociation *in vitro* assays (Figure 3) unequivocally confirm this hypothesis, showing that, in the presence of colchicine, the TBCB/TBCE dissociation machine is unable to dissociate the tubulin heterodimer (Figure 3). Given these results, the decrease in the amount of TBCA/β-tubulin complex during the colchicine treatment is easily explained by the absence of β-tubulin that cannot be released by tubulin cofactors from the dimer, resulting in the detection of free TBCA.

Colchicine and nocodazole are both microtubule binding drugs that targets the β-tubulin subunit at a pocket close to the α-tubulin/β-tubulin heterodimer interface. It has been proposed that both compounds disrupt the MT dynamics by preventing the curve-to-straight conformational change of the α/β-tubulin heterodimer upon GTP hydrolysis (Wang et al., 2016). Our structural prediction of the α/β-tubulin-colchicine 3D structure associated with TBCB/TBCE (Figure 4), indicates that, in the presence of the colchicine, the α/β-tubulin interface might adopt a closer and more stable conformation, able to resist the mechanical force applied by the LRR domain of TBCE, which could explain the inability of TBCB/TBCE to dissociate the tubulin heterodimer. In the case of nocodazole, the molecule is structurally very different of colchicine and show different binding dynamics. Firstly, the nocodazole molecule is placed deeper in the β-tubulin monomer than the colchicine and it does not seem to interact with the α-tubulin monomer (Aguayo-Ortiz et al., 2013; Wang et al., 2016). Secondly, while colchicine is irreversibly bound to the β-tubulin and its effects on MT polymerization is also irreversible; nocodazole is a rapidly reversible inhibitor. Because the α/β-tubulin heterodimer conformation in the presence of colchicine is very similar to that in the presence of nocodazole, it is tempting to think that the nocodazole bound-heterodimer would be also resistant to the TBCE, TBCB dissociation activity. However, nocodazole results are distinct from those originated by colchicine (Figure 1). It is plausible that the resistance to the mechanical force applied to the heterodimer by the tubulin cofactors might require a more stable drug binding-state of it to be evaluated. Although the α/β-tubulin heterodimer dissociation kinetics remains to be quantified, it is reasonable to propose that the dissociation activity of the TBCE and TBCB cofactors may be slower than the binding kinetics of nocodazole. In bulk, the dissociation activity taking place on free heterodimers would hinder detection of the activity on nocodazole bound heterodimers.

It was known that in *in vitro* tubulin dissociation assays performed in the presence of TBCB and TBCE, but in the absence of TBCA, β-tubulin becomes unstable and aggregates (Kortazar et al., 2006). The formation of those aggregates might be avoided by the addition of TBCA (Kortazar et al., 2006). These results motivated us to investigate how excess or deficiency in TBCA levels affect tubulin heterodimer dissociation by analyzing the levels of the TBCA/β-tubulin complex. Interestingly, and contrary to our prediction, TBCA overexpression leads to a decrease of the α/β-tubulin heterodimer levels (Figure 5A). Strikingly, TBCA depletion (Figure 5C) also causes the decrease of α/β-tubulin heterodimer (Nolasco et al., 2005) and present work). TBCA overexpression results support the evidence that TBCA plays an important role in the recycling pathway of the tubulin heterodimer. Indeed, excess TBCA does not lead to an increase in tubulin dimerization. The observed decrease of α/β-tubulin heterodimer amounts, together with the increase in the TBCA/β-tubulin complex, strongly suggests that, when more TBCA is available, the tubulin heterodimer proceeds through the recycling route. In the case of cells treated with TBCA siRNA, the diminished levels of the α/β-tubulin heterodimer are possibly the consequence of driving tubulin to degradation, since the recycling pathway cannot be completed due to low TBCA levels. Therefore, it is conceivable that, *in vivo*, TBCA concentration determines tubulin fate being it recycling or degradation. It was proposed that the UBL domains of both TBCE and TBCB in the dissociation complex remain free and exposed, protruding from opposite positions in the central mass of the complex (Serna et al., 2015). This observation strongly suggests that both domains could be involved in the degradation of α-tubulin subunit by the proteasome, while no degradation mechanism was previously described for β-tubulin. In our study we show that β-tubulin is degraded in cells depleted of TBCA, indicating that TBCD, known to dissociate the α/β-tubulin heterodimer by itself and to stabilize the β-tubulin (Martín et al., 2000), does not seem to have a prominent role in its recycling pathway.

In the last years, numerous studies have shown that altered levels of TBCA expression are associated with human diseases like cancer, neurodegenerative diseases, osteoporosis, and Ankylosing spondylitis (Table 1). Strikingly the human diseases associated with the other TBCs are mainly due to mutations in genes encoding these proteins (review Serna and Zabala, 2016). According to our results, the altered levels of TBCA will affect the α/β-tubulin heterodimer recycling/degradation, which will impact MT cytoskeleton dynamics and remodeling. Nothing is known about the role of TBCs in the incorporation of distinct tubulin isotypes in different MT classes, or alternatively in response to specific signals. We envisage that the recycling pathway of tubulins will have a prominent role in altering the biochemical/biophysical properties of MTs by allowing the polymerization of different tubulin isotypes.

Our results also show that TBCA is enriched in insoluble protein extracts obtained from adult heart mice (Figure 6). At first glance, this observation is unexpected, but a detailed analysis of the cardiomyocyte cytoskeleton reveals that MTs in crosstalk with actin and intermediate filaments play specific roles in sustaining the cardiac contractile function (Kuznetsov et al., 2020). In cardiomyocytes, MTs are known to be involved in the intracellular traffic and the transmission of mechanical signals, in the shaping of membrane systems, and in the spatial organization of myofibrils and organelles (Caporizzo et al., 2019). Probably, the demanding roles of MTs in these cells, such as mechanical transduction, may require intense recycling of tubulin heterodimers, explaining the abundance of TBCA. In fact, it was recently shown that MTs exchange tubulin dimers at lattice defects sites induced by mechanical stress (Schaedel et al., 2015; Aumeier et al., 2016). Indeed, TBCD was downregulated by arterial shear stress (Schubert et al., 2000). These events may occur spontaneously during MT polymerization due to MT-severing enzymes or other unknown factors (Schaedel et al., 2015; Aumeier et al., 2016). The specific roles of TBCs, including the role of TBCA shown in this work, indicate that these molecules are excellent candidates to be involved in these stress responses.

It is still intriguing the observation of high levels of TBCA in the insoluble protein extracts of the heart tissue. In cardiac cells, MTs are located close to the nuclear envelope, in the transverse (T)-tubules membrane, and at the mitochondrial membrane (Caporizzo et al., 2019). It was observed that tubulin heterodimers are bound to and regulate the voltage-dependent anion channel (VDAC) in mitochondria (Carré et al., 2002). This attachment that seems to be established through a specific β-tubulin isotype (βII-tubulin), is strong enough to maintain β-tubulin in an ordered striated distribution pattern that colocalizes with the mitochondria, even after MT depolymerization and cells permeabilization (Rappaport and Samuel, 1988; Guerrero et al., 2010). Moreover, it was described that in cardiomyocytes, about 70% of total tubulin is present in MTs, whereas 30% occurs as free tubulin heterodimers (Tagawa et al., 1998; Hein et al., 2000; Kostin et al., 2000). Therefore, we can speculate that TBCA may be associated with β-tubulin in these localizations playing an unknown function, which could justify the enrichment of TBCA in the insoluble fraction. Alternatively, the canonical role of TBCA/β-tubulin complex in β-tubulin recycling may be performed *in situ* in close association with membranes.

Colchicine is a therapeutical hallmark in pericardial diseases characterized by densification of the MT network, leading to increased cell stiffness (Hein et al., 2000). It has been assumed that colchicine has a beneficial role in cardiomyocytes via decreasing MT mass and altering MT dynamics (Hein et al., 2000). Our work shows, for the first time, that colchicine affects the tubulin recycling process by inhibiting tubulin heterodimer dissociation, which leads to the decrease in the amount of TBCA/β-tubulin complex, resulting in free TBCA in the cell. As such, our results open the possibility that TBCA/β-tubulin complex may have unknown roles in cardiomyocytes. Future studies are required to elucidate the consequences of free TBCA in cells and overall colchicine mechanism of action.

From this work, TBCA emerges as an important protein in the recycling/degradation of β-tubulin and, consequently, in the regulation of MT dynamics. Indeed, this idea is strongly supported by the fact that TBCA has been associated with different human diseases where its levels are altered (Table 1). Although providing an important contribution, our results suggest that the role of TBCA and/or TBCA-tubulin complex’s role in eukaryotic cells require further validation from *in vitro*, and, most importantly, *in vivo* studies.

## Materials and Methods

### Cell Culture

HeLa human tumor cell line, was cultured in a 5% CO_2_ humidified atmosphere at 37°C as exponentially growing sub-confluent monolayers in Dulbecco’s modified Eagle’s medium (DMEM) with Glutamax (Invitrogen; Thermo Fisher Scientific, Inc., Waltham, MA, United States), supplemented with 10% fetal calf serum (Invitrogen; Thermo Fisher Scientific, Inc., Waltham, MA, United States) and non-essential amino acids (Invitrogen; Thermo Fisher Scientific, Inc., Waltham, MA, United States).

The MT depolymerizing experiments were done after 15, 30, and 60 min incubation with either 5 μM colchicine (Sigma, St. Louis, United States), or 30 μM nocodazole (Sigma, St. Louis, United States) or with cold shock (culture media at 4°C was added to the cells and the cell plates were put on ice during the treatment (Weisenberg, 1972; Shelanski et al., 1973). The concentrations of colchicine and nocodazole used in this work aimed at causing an accentuated and rapid increase of tubulin heterodimers due to MT depolymerization and are similar to those already used in other cell lines (Breitfeld et al., 1990; Karbowski et al., 2001). Translation inhibition was carried out by the cycloheximide (Sigma, St. Louis, United States) treatment (50 μg/ml).

TBCA siRNA and TBCA overexpression transfection experiments were performed according to our previous results (Nolasco et al., 2005; Kortazar et al., 2007).

### Protein Extraction From HeLa Cells

For native protein extracts, cells were rinsed twice with PBS. During the second PBS wash, cells were removed from culture plates by scraping and then centrifuged at 3,500 *g* for 5 min at room temperature (RT). Then, PBS was discarded, and the cell pellets were resuspended in lysis buffer (100 mM MES, pH 6.7 (Sigma, St. Louis, United States); 1 mM MgCl_2_ (Merck, Darmstadt, Germany); 1 mM EGTA (Sigma, St. Louis, United States); 0.1 mM GTP (Sigma, St. Louis, United States); 0.2 mM DTT (Sigma, St. Louis, United States) and 0.1 mM PMSF (Sigma, St. Louis, United States), containing a protease inhibitors cocktail (Thermo Fisher Scientific, Inc., Waltham, MA, United States). Total protein extracts were obtained by homogenizing samples at room temperature or 4°C mechanically, using a potter-homogenizer. Cellular lysates were centrifuged at 14,000 *g* for 30 min at RT. The supernatants were recovered (soluble fraction). An important note is that, except for samples submitted to cold shock, all protein extracts were prepared at RT and RT solutions/buffers to avoid MT depolymerization due to cold solutions/buffers normally used to prevent protein degradation.

After protein quantification by Bradford (Bio-Rad, California, United States), equal amounts of soluble protein extracts (30 μg) were separated on non-denaturing gel electrophoresis [6% (w/v) Native-PAGE] (Zabala and Cowan, 1992; Fontalba et al., 1993). Protein extracts from TBCA siRNA and TBCA overexpression experiments were also analyzed on a 16.5% (w/v) Tricine–SDS–PAGE (Schägger and von Jagow, 1987).

### Protein Extraction From Different Mouse Organs

Mouse organs were pulverized cryogenically in liquid nitrogen using a mortar and pestle for grinding frozen tissue. 200 μl of lysis buffer [1% NP40; 50 mM HEPES, pH 8 (Sigma, St. Louis, United States); 200 mM NaCl (Sigma, St. Louis, United States); 5 mM EDTA (Sigma, St. Louis, United States); 0.2 mM DTT (Sigma, St. Louis, United States) and 0.1 mM PMSF (Sigma, St. Louis, United States)], containing a protease inhibitors cocktail (Thermo Fisher Scientific, Inc., Waltham, MA, United States) were added to 150 mg of the pulverized organ and homogenized using a potter-homogenizer. Cellular lysates were removed and centrifuged at 14,000 × *g* for 90 min at 4°C. After protein quantification by Bradford (Bio-Rad, California, United States), equal amounts of soluble protein extracts (100 μg) were separated on 16.5% (w/v) Tricine–SDS–PAGE (Schägger and von Jagow, 1987). 100 μl of 8 M urea were added to the pellets of the insoluble fraction. The insoluble protein extracts were diluted 1:2 in loading buffer, and 20 μl were analyzed on 16.5% (w/v) Tricine–SDS–PAGE (Schägger and von Jagow, 1987).

### Western Blot Analysis

Westerns blots were performed using the rabbit polyclonal serum against TBCA (1:5,000) (Llosa et al., 1996), the mouse monoclonal antibody against β-tubulin (1:1,000) (clone TUB 2.1, Sigma, St. Louis, United States), the mouse monoclonal antibody against α-tubulin (1:1,000) (clone DM1A, Sigma, St. Louis, United States) and the rabbit monoclonal antibody against β-actin (1:2,000) (Sigma, St. Louis, United States). Secondary antibodies against rabbit and mouse (Jackson Immuno Research) were used at 1:4,000. The immunostaining was carried out using the ECL technique (GE Healthcare). The presented results are representative of at least three independent experiments.

### Fluorescence Microscopy

For immunofluorescence microscopy studies, cells were washed twice with PBS (Invitrogen; Thermo Fisher Scientific, Inc., Waltham, MA, United States) and then fixed in 3.7% (w/v) paraformaldehyde (Merck, Darmstadt, Germany) in PBS for 10 min at room temperature. They were then washed twice with PBS for 5 min and permeabilized for 2 min using 0.1% (v/v) Triton X-100 (Sigma, St. Louis, United States) in PBS. After rinsing twice for 5 min in PBS and once in PBS-0.1% (v/v) Tween-20 (Merck, Darmstadt, Germany), cells were blocked in 3% (w/v) BSA (Calbiochem; Sigma, St. Louis, United States) for 15 min. Next, cells were incubated with the mouse monoclonal α-tubulin antibody (clone DM1A, Sigma, St. Louis, United States) at 1:200 in the same solution for 60 min. Samples were washed twice in PBS for 5 min and once in PBS-0.1% (v/v) Tween-20. Secondary antibody Alexa Fluor 488-conjugated goat anti-mouse IgG at 1:500 (Molecular Probes; Invitrogen; Thermo Fisher Scientific, Inc., Waltham, MA, United States) was incubated for 60 min in the same solution. The preparations were washed twice in PBS for 5 min and once in PBS-0.1% (v/v) Tween-20. DNA was stained with DAPI (1 μg/μl) in PBS for 1 min. The preparations were washed in PBS and mounted in MOWIOL 4-88 (Calbiochem; Sigma, St. Louis, United States) mounting medium supplemented with 2.5% (w/v) DABCO (Sigma, St. Louis, United States). Cells were examined with a fluorescence microscope (Leica DMRA2), and image acquisition was performed with a cooled CCD camera and MetaMorph Imaging Software (Universal Imaging Corporation). Image processing was carried out with ImageJ Software.

### Protein Production for *in vitro* Assay

Human TBCA was cloned and purified from *Escherichia coli* cells (Llosa et al., 1996). Human TBCE and TBCB were purified from insect cells infected with recombinant baculovirus and *E. coli* cells, respectively (Kortazar et al., 2007). Tubulin dimers were isolated and purified from bovine brain (Avila et al., 2008).

### Tubulin Purification After Colchicine Incubation

The *in vitro* assay of colchicine binding to tubulin was done under conditions where the drug was present in large excess over tubulin (Banerjee and Luduena, 1992). Bovine brain purified tubulin (60 μM) was incubated with colchicine (1.4 mM) for 1 h at room temperature, and tubulin-colchicine heterodimers were purified by gel filtration (Superdex 200 PC 3.2/30) using a Smart System (Amersham Pharmacia Biotech) to remove unbounded colchicine. The elution buffer used was 0.1 M MES pH 6.7, 1 mM MgCl_2_, 1 mM EGTA, 25 mM KCl and 0.1 mM GTP. Fractions of 50 μl were eluted at 40 μl/min. As a control, the same amount of bovine brain purified tubulin was also incubated for 1 h at room temperature, without colchicine, followed by gel filtration purification. Each peak of tubulin and tubulin-colchicine heterodimers were pooled (17–19 fractions).

### Tubulin Dimer Dissociation Assay

Purified tubulin and tubulin-colchicine heterodimers, from pooled fractions (2.2 μg), were incubated at 30°C for 30 min with or without TBCE (1.5 μg/reaction), TBCB (0.6 μg/reaction) and TBCA (5 μg/reaction) in buffer 0.1 M MES pH 6.7, 1 mM MgCl_2_ and 0.1 mM GTP, to perform tubulin heterodimer dissociation assays. These assays were analyzed on 6% (w/v) native-PAGE (Zabala and Cowan, 1992; Kortazar et al., 2007) and stained with Coomassie brilliant blue.

### Statistical Analysis

The experiments were performed at least three times and the results were expressed as means ± S.D. Differences between the data were tested for statistical significance by t-test. *P* < 0.05 were considered statistically significant.

## Data Availability Statement

The original contributions presented in the study are included in the article/Supplementary Material, further inquiries can be directed to the corresponding author/s.

## Ethics Statement

The animal study was reviewed and approved by the Comite de Bioetica de la Universidad de Cantabria.

## Author Contributions

SN: conceptualization, resources, funding acquisition, data curation (molecular and cell biology and transfection, fluorescence microscopy, western blot), image composition, and writing (original draft, review and editing). JB: conceptualization, resources, and data curation (protein purification, protein analysis, westerns, antibodies production, and purification). MS and BC: data curation and analysis, writing (original) image, and table composition. HS and JZ: conceptualization, resources, funding acquisition, and writing (original draft, review and editing). All authors contributed to the article and approved the submitted version.

## Conflict of Interest

The authors declare that the research was conducted in the absence of any commercial or financial relationships that could be construed as a potential conflict of interest.

## Footnotes

1 https://clinicaltrials.gov/ct2/results?cond=COVID&term=colchicine&cntry=&state=&city=&dist
