# Supplementary material for: Colchicine Blocks Tubulin Heterodimer Recycling by Tubulin Cofactors TBCA, TBCB, and TBCE
Source: Front Cell Dev Biol. 2021 Apr 22;9:656273. doi: 10.3389/fcell.2021.656273 (PMC8100514; doi:10.3389/fcell.2021.656273)
Supplement: Supplementary file 1 [file Table_1.DOCX]

Supplementary Material

# Supplementary Figures


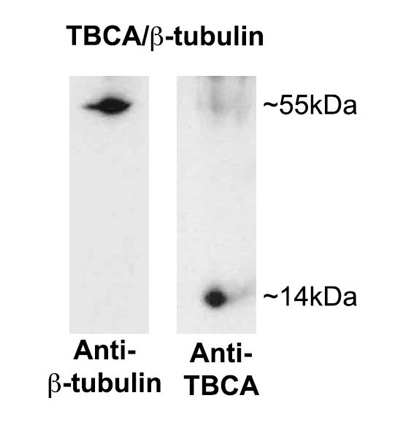


**Supplementary Figure 1. TBCA/**$\boldsymbol{\beta}$**-tubulin analysis on non-classical 2D electrophoresis.** Soluble protein extracts from HeLa cells (30 $\mu$g) were analyzed by non-denaturing gel electrophoresis (6% (w/v) Native-PAGE) stained with Coomassie brilliant blue. The TBCA/β-tubulin complex band was excised and loaded onto a 16.5% Tricine–SDS PAGE and analyzed by western-blot using, sequentially, the specific antibodies to β-tubulin and TBCA. These results clearly show the presence of both proteins in this complex. The approximate molecular mass of the proteins is indicated on the right side of the panels.


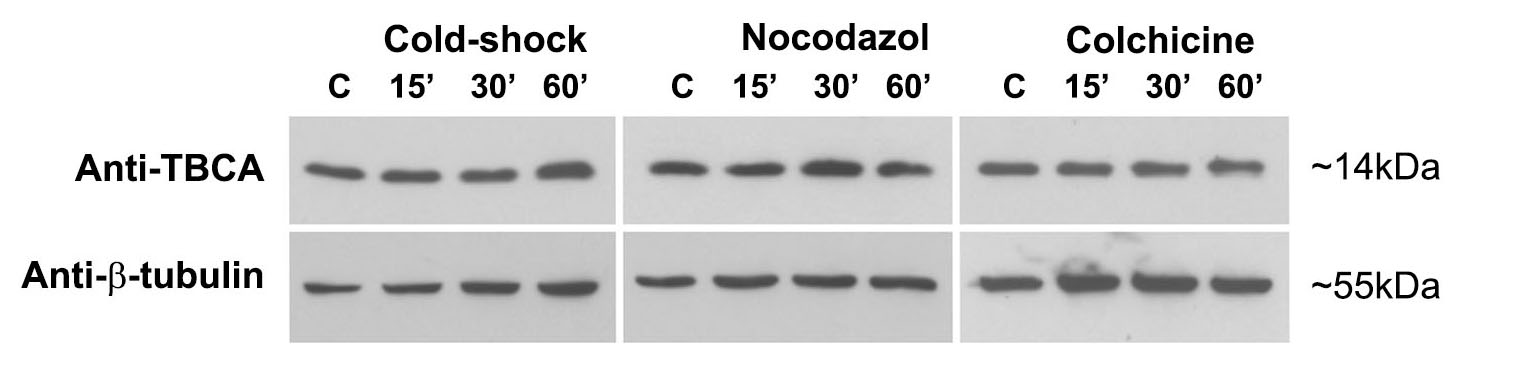


**Supplementary Figure 2.** Denaturing analysis of HeLa protein extracts treated with MT depolymerizing agents (Colchicine, Nocodazole and Cold Shock). In parallel with the native-PAGE analysis (see Figure 1), as a loading control, the same extracts were analyzed in 16.5% Tricine–SDS–PAGE followed by western blot with antibodies directed to TBCA and $\beta$-tubulin. The results showed that TBCA levels did not change during the referred treatments, while $\beta$-tubulin levels slightly increase. The approximate molecular mass of the proteins is indicated on the right side of the panels.


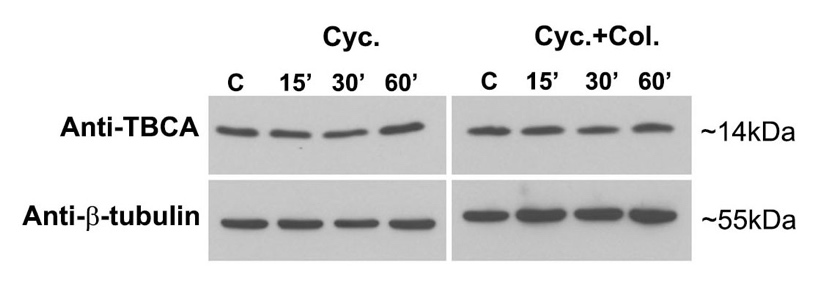


**Supplementary Figure 3.** Denaturing analysis of protein extracts from HeLa cells treated with cycloheximide and cycloheximide plus colchicine. In parallel with the native-PAGE (see Figure 2), as a loading control, the same extracts were analyzed in 16.5% (w/v) Tricine–SDS–PAGE followed by western blot with antibodies directed to TBCA and $\beta$-tubulin. The results showed that TBCA levels did not change during the referred treatments, while $\beta$-tubulin levels slightly increase in the presence of colchicine. The approximate molecular mass of the proteins is indicated on the right side of the panels.

**
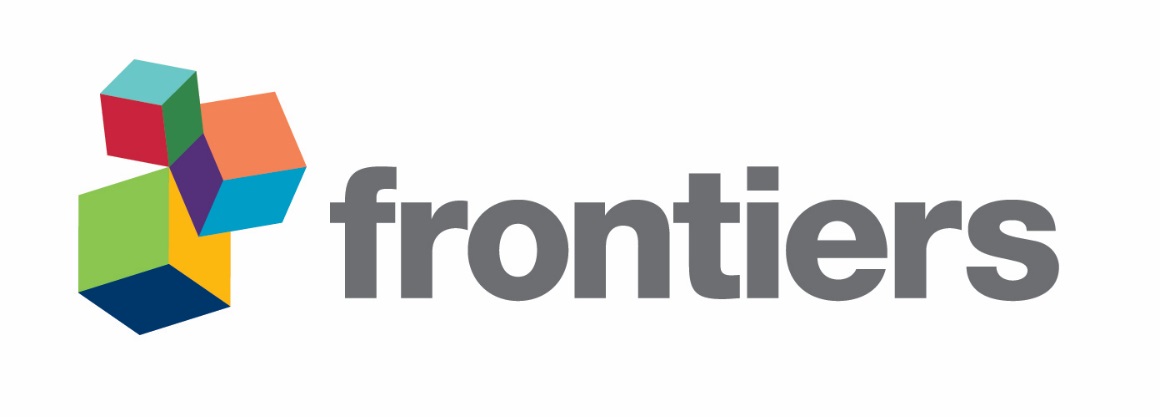
**
